# From network hub to therapeutic target: the role of mediodorsal thalamic nucleus in epilepsy

**DOI:** 10.3389/fnins.2026.1871447

**Published:** 2026-06-17

**Authors:** Haipeng Xie, Wenling Li

**Affiliations:** 1 Affiliated Hospital of Hebei University, Baoding, Hebei, China; 2 Second Hospital of Hebei Medical University, Shijiazhuang, Hebei, China

**Keywords:** brain networks, cognitive functions, epilepsy, mediodorsal thalamic nucleus, neuromodulation

## Abstract

Mediodorsal thalamic nucleus (MD) is a pivotal hub for cortical functions, characterized by significant heterogeneity in its anatomical connectivity, cytoarchitecture, and function, constituting a complex nucleus composed of multiple functionally specialized subregions. We elaborates on the heterogeneous anatomical connectivity of MD and its crucial role in supporting higher cognitive functions such as working memory, cognitive control, and emotional integration. It then focuses on the multifaceted role of MD within epileptic pathological networks. Substantial evidence indicates that MD in patients with epilepsy exhibits structural atrophy and abnormalities in functional connectivity, with its activity being recruited early during seizures and likely involved in seizure propagation and generalization. However, the therapeutic efficacy of neuromodulation targeting MD, such as deep brain stimulation (DBS), remains contentious, highlighting the current insufficient understanding of its distinct functional subregions and specific pathway mechanisms. Finally, the review discusses the challenges and future directions in translating MD into an effective therapeutic target. It emphasizes that future research must endeavor to elucidate its causal mechanisms within epileptic networks at the subregional level, account for the heterogeneity of seizure onset frequencies, and develop precise intervention strategies targeting specific epileptogenic pathways, thereby advancing novel therapies focused on thalamocortical circuits toward clinical application.

## 1 Introduction

Mediodorsal thalamic nucleus (MD) is a central hub for prefrontal cortical function. Traditionally, MD has often been viewed as a homogeneous entity. However, accumulating evidence indicates that MD exhibits high degrees of heterogeneity in its anatomical connectivity, cytoarchitecture, and function, constituting a complex nucleus composed of multiple functionally specialized subregions. This organizational specificity dictates its ability to support varied high-level cognitive processes and governs its involvement pattern and therapeutic relevance in brain network diseases, including epilepsy.

**Figure 1:**
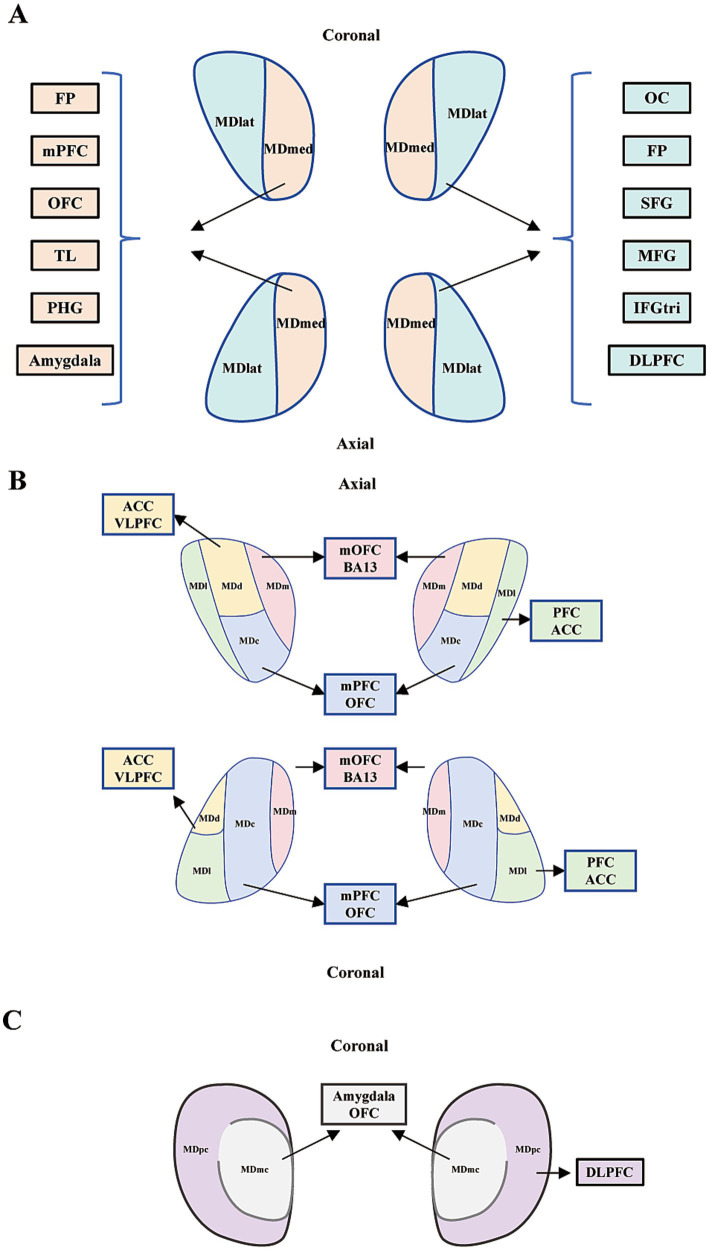
Three parcellation methods of the mediodorsal thalamic nucleus (MD) and the characteristics of cortical connectivity for each subregion. **(A)** Jakab et al. parcellated the MD into a medial (MDmed) and a lateral (MDlat) subdivision. MDmed is predominantly connected to the medial frontal cortex and limbic system, while MDlat shows stronger connectivity with the lateral frontal cortex (Jakab et al., 2012). **(B)** Li et al. subdivided MD into four regions: MDm, which is connected to the medial orbitofrontal cortex and BA13; MDc, which exhibits stronger connectivity with the medial frontal cortex and orbitofrontal cortex; MDd, which is closely associated with the ventrolateral prefrontal cortex and anterior cingulate cortex; and MDl, which is connected to the prefrontal and anterior cingulate cortices (Li et al., 2022). **(C)** Based on its cytoarchitectonic organization, MD can be subdivided into distinct regions. MDmc is predominantly connected to the amygdala and orbitofrontal cortex, while MDpc is extensively connected to the dorsolateral prefrontal cortex (Chakraborty et al., 2019). ACC, Anterior Cingulate Cortex; DLPFC, Dorsolateral Prefrontal Cortex; FP, Frontal Pole; IFGtri, Pars Triangularis of the Inferior Frontal Gyrus; mPFC, Medial Prefrontal Cortex; MFG, Middle Frontal Gyrus; mOFC, Medial Orbitofrontal Cortex; OFC, Orbitofrontal Cortex; OC, Occipital Cortex; PFC, Prefrontal Cortex; PHG, Parahippocampal Gyrus; SFG, Superior Frontal Gyrus; TL, Temporal Lobe; VLPFC, Ventrolateral Prefrontal Cortex.

Anatomically, the human MD can be parcellated into distinct subregions based on its topographic connectivity with the prefrontal cortex. For instance, connectomic studies utilizing diffusion magnetic resonance imaging have revealed that the MD can be subdivided into a medial part (MDmed) and a lateral part (MDlat), which are predominantly connected to the orbitofrontal/medial prefrontal cortex and the dorsolateral prefrontal cortex, respectively (Jakab et al., 2012). More refined parcellation schemes have further delineated four subregions: medial (MDm), central (MDc), dorsal (MDd), and lateral (MDl). These subregions exhibit specific, robust connectivity with distinct cortical areas, namely the medial orbitofrontal, ventrolateral prefrontal, and dorsolateral prefrontal cortices (Li et al., 2022). This ordered connectivity topography provides the anatomical basis for the functional specialization of MD (Figure 1).

Different subregions of the MD support distinct cognitive and affective functions. The magnocellular subdivision (MDmc), which primarily receives inputs from limbic structures such as the amygdala and is connected to the orbitofrontal cortex, plays a key role in emotional integration, reward-related decision-making, and familiarity-based recognition memory (Benarroch, 2021; Chakraborty et al., 2019). Given its tight coupling with the dorsolateral prefrontal cortex, the parvocellular subdivision (MDpc) serves as a key substrate for working memory and higher-order executive functions, notably cognitive flexibility and adaptive decision-making (Chakraborty et al., 2019; Parnaudeau et al., 2013). Collectively, this evidence establishes that the MD is not a functionally homogeneous structure. Rather, its medial (predominantly engaging limbic-affective circuits) and lateral (predominantly engaging cognitive-executive circuits) subregions play divergent roles in information processing and cognitive control (Pergola et al., 2018).

It is precisely the MD’s role as a hub, integrating information from the limbic system and the neocortex, that may make it a critical node for the propagation of epileptiform activity. The dense bidirectional connections between the MD and structures such as the hippocampus, amygdala, anterior cingulate cortex, and extensive prefrontal cortical regions form a potential “divergent-convergent” excitatory amplification system (Benarroch, 2021; Timbie and Barbas, 2015; Sloan et al., 2011). In the pathological context of epilepsy, this circuitry, which is normally dedicated to integrating higher-order cognitive information, may be hijacked to serve as a conduit for seizure generalization. Clinical studies have found that in patients with conditions such as temporal lobe epilepsy, MD exhibits structural atrophy and abnormalities in functional connectivity, and its involvement is closely associated with both seizure frequency and contralateral spread (Marais et al., 2025; Jo et al., 2019; Keller et al., 2012; Wu et al., 2023). Animal studies have further confirmed that inhibition of specific MD-prefrontal cortex pathways can effectively control seizures, whereas nonspecific electrical stimulation of the MD nucleus itself yields no therapeutic effect (Wicker et al., 2022; Wang et al., 2010).

In summary, the mediodorsal thalamic nucleus (MD), the central hub of prefrontal function, is characterized by high complexity and heterogeneity in both its structure and function. This inherent complexity enables it to serve as an integrator in normal cognition, while also potentially acting as a propagation node for epileptic network activity under pathological conditions. However, there remains a lack of systematic elaboration on several key issues: the specific mechanisms by which the MD contributes to epilepsy, how the interplay between its altered structural connectivity and functional activity leads to cognitive comorbidities and the formation of seizure networks, and how it can be translated into an effective therapeutic target. Therefore, this article aims to synthesize the anatomical connectivity and functional organization of MD, with a focus on its subregional heterogeneity; to examine its specific pathological roles in epilepsy, encompassing structural changes, functional connectivity abnormalities, and network dynamics; and to evaluate the current evidence, controversies, and future directions for MD-targeted neuromodulation strategies. By integrating basic research with clinical evidence, this review aims to provide a clear framework for a deeper understanding of the pivotal role of the MD in epileptic networks and to clarify the differential contributions of its distinct components. Furthermore, it seeks to outline a direction for developing novel, precise therapeutic strategies targeting thalamocortical circuits.

## 2 Projection fibers and network connectivity

### 2.1 Receptors

Cholinergic receptors are present within MD. Three-dimensional reconstruction reveals that these receptors are arranged in a cylindrical structure along the rostrocaudal axis of MD nucleus (Brandel et al., 1990). In human thalamus, the density of nicotinic acetylcholine receptors in MD is significantly higher than in all other thalamic subregions (Garibotto et al., 2020). The ventral and lateral portions of the mediodorsal thalamic nucleus possess the highest density of dopaminergic receptors. In contrast, noradrenergic receptors are uniformly distributed throughout the mediodorsal thalamic nucleus (Melchitzky and Lewis, 2001). MD and basolateral amygdala (BLA) send convergent projections to medial prefrontal cortex. Neurons projecting from BLA to MD include not only glutamatergic neurons but also long-range GABAergic neurons. The differential recruitment of these two neuronal populations may influence the coordination of activity between BLA, MD, and medial prefrontal cortex (Ahmed and Paré, 2023). Metabotropic glutamate receptors modulate cognitive circuits by regulating neuronal activity patterns in MD, and this modulatory effect exhibits heterogeneity across different thalamic nuclei (Copeland et al., 2015). MD has extensive bidirectional connections with prefrontal cortex (PFC). Both PFC-MD and MD-PFC pathways are glutamatergic and excitatory. Cortical excitation arriving at MD is not only relayed back to MD itself via deep pyramidal cells but is also transferred to contralateral prefrontal cortex via layer III pyramidal cells in ipsilateral prefrontal cortex. Fibers projecting from ventral pallidum and substantia nigra pars reticulata to MD are primarily GABAergic inhibitory neurons (Kuroda et al., 1998).

### 2.2 Fiber connectivity

MD receives extensive afferent inputs from multiple brain regions, including septal area, amygdaloid complex, hypothalamus, mammillary bodies, olfactory tubercle, claustrum, ventral mesencephalic area, zona incerta, substantia nigra, periaqueductal gray, and the mesencephalic reticular formation (Gritti et al., 1987). MDmc receives fiber projections from amygdala and is directly connected to the orbitofrontal cortex, forming a tightly interconnected tripartite network (Timbie and Barbas, 2015). Fibers connecting MD to frontal cortex travel within anterior limb of internal capsule (Kito et al., 2009). This connectivity exhibits distinct spatial topography, with fibers linking to dorsolateral prefrontal, ventrolateral prefrontal, and orbitofrontal cortices organized in a dorsoventral arrangement (Jang and Yeo, 2014). Overall, strong connectivity exists between medial MD and the lateral orbitofrontal cortex, dorsocaudal MD and medial frontal/cingulate cortex, and lateral MD and lateral prefrontal cortex (Klein et al., 2010). Both anterior thalamic nucleus (ANT) and MD show high connectivity with the prefrontal cortex; however, MD demonstrates stronger fiber connectivity with primary motor and premotor cortices compared to ANT (Jang S. H. et al., 2018).

In the transverse axis of the MD, an orderly topographic gradient exists for MD-PFC projection areas: the anteromedial pole of MD preferentially connects with ventromedial and orbital PFC regions, while the posterolateral pole of MD preferentially connects with posterolateral PFC regions, with intermediate PFC regions occupying the intervening territory (Phillips et al., 2019). A study by Jakab et al., using connectivity-based gray matter parcellation, revealed two substructural domains within human mediodorsal thalamus: a medial part (MDmed) and a lateral part (MDlat). The separation plane for these two regions runs parallel to the midline, resulting in medial and lateral partitions of the mediodorsal nucleus. MDlat is primarily connected to the lateral occipital cortex, frontal pole, superior frontal gyrus, anterior part of the middle frontal gyrus, pars triangularis of the inferior frontal gyrus, and supplementary motor area (dorsolateral prefrontal cortex). In contrast, MDmed is mainly connected to the frontal pole, mPFC, orbitofrontal cortex, temporal lobe, anterior parahippocampal gyrus, and amygdala (Jakab et al., 2012). Research by Li et al. further subdivided human MD into four subregions: MDm (medial), MDc (central), MDd (dorsal), and MDl (lateral). Among these, MDm is primarily connected to the medial orbitofrontal cortex, and its connectivity with BA13 is significantly stronger than that of other subregions. MDc shows stronger connectivity with parts of the medial frontal cortex and orbitofrontal cortex. MDd is connected to most ventrolateral prefrontal cortex regions and has strong links with the anterior cingulate cortex and BA46. MDl has multiple projections to the prefrontal and anterior cingulate cortices (Li et al., 2022). Human mediodorsal thalamic nucleus (MD) exhibits a defined pattern of preferential cortical connectivity, demonstrating stronger structural links with the pregenual anterior cingulate cortex, dorsal anterior cingulate cortex, dorsolateral prefrontal cortex, and the caudate nucleus. These specific connectivity profiles underpin the central role of the MD within cognition-limbic circuitry. Furthermore, these connections align with the classic dorsolateral prefrontal-thalamo-striatal circuit, which is implicated in higher-order cognition, motivation, and motor planning. Additionally, the robust connectivity between the MD and the cingulate cortex situates it within the limbic system network, facilitating its involvement in emotional and motivational processing (Eckert et al., 2012).

### 2.3 Functional brain networks

The thalamus nucleus serve as primary cognitive integration regions and convergence points between different cortical cognitive networks, including the default network, ventral attention network, salience network, and frontoparietal control network. MD is interconnected with the core cortical components of these networks, encompassing the ventromedial prefrontal cortex (default network), ventrolateral prefrontal cortex (ventral attention network), anterior cingulate cortex (salience network), and dorsal prefrontal cortex (frontoparietal control network) (Benarroch, 2021). In the study by Li et al., MDm demonstrated stronger functional connectivity with higher-order cortical networks such as default network and frontoparietal network, whereas the functional connectivity profiles of the other three subregions with the seven networks were relatively uniform. The strength of functional connectivity between MD subregions and visual, somatomotor, ventral attention, and dorsal attention networks showed an increasing trend from MDm to MDc, MDd, and MDl. All four subregions showed significant connectivity with default network and frontoparietal network, with MDd exhibiting the strongest connectivity to default network. The connectivity of MDd and MDl to frontoparietal network was significantly stronger than that of the other two subregions (Li et al., 2022). Functional connectivity between MD and default mode network (DMN) may play a significant role in the pathogenesis of chronic disorders of consciousness (DOC). Compared to healthy controls, DOC patients show a significant reduction in functional connectivity between MD and brain regions within the DMN, including the medial prefrontal cortex and posterior cingulate cortex/precuneus (He et al., 2015). The integrity of MD-DLPFC connectivity serves as a significant independent predictor of motor functional prognosis in patients with damage to descending motor pathways. When the primary descending motor pathways are interrupted, preserved MD-DLPFC connectivity can support superior compensatory motor recovery. Conversely, concurrent damage to both the corticospinal tract and the MD-DLPFC connection is associated with poorer motor recovery. In summary, the MD-DLPFC connection constitutes a critical “reserve” or alternative circuit for post-stroke motor recovery, whose significance becomes particularly salient following injury to the primary motor pathways (Choi et al., 2021).

## 3 Cognitive functions

### 3.1 Memory and learning

Functional connectivity exists between the entorhinal cortex, parahippocampal gyrus, and perirhinal cortex with medial, orbital, and lateral PFC (Kafkas et al., 2020). This functional connectivity, centered on MD, participates in familiarity-based recognition memory processes through synchronized beta oscillations (Benarroch, 2021). It also plays a crucial role in spatial long-term memory (Spets and Slotnick, 2020). During the acquisition and execution of working memory tasks, synchrony in the beta frequency band between MD and PFC is enhanced (Parnaudeau et al., 2013). Even subtle decreases in MD activity are sufficient to cause memory dysfunction. A case reported by Ellis et al. described a patient with a right-sided MD lesion who exhibited deficits in the familiarity-based recognition of new faces, while word recall remained unaffected (Edelstyn et al., 2016). Patients with lesions to MDpc show reduced picture recognition ability, manifested as decreased recall volume and prolonged reaction times for pictures (Pergola et al., 2012). In contrast, patients with medial MD lesions often exhibit impaired prospective memory (Cona et al., 2019). Using diffusion tensor tractography (DTI), Jang et al. were the first to directly observe and confirm damage to the MD-OFC connection in a patient with traumatic brain injury (TBI), a finding that correlated with the patient’s memory impairment. This case demonstrates that the MD-OFC pathway is a key circuit supporting memory function, and its damage may serve as a potential imaging biomarker for specific types of memory deficits following TBI (Jang S. et al., 2018). MD does not support a single cognitive function in isolation but acts as a critical regulatory hub. Its core function lies in modulating activity patterns in brain regions such as the prefrontal cortex, particularly during complex cognitive processes that require the maintenance and prolongation of sustained prefrontal neural activity to cope with prolonged delays, interference, or multitasking. Disruption of the MD-PFC circuit underpins cognitive deficits in a variety of neurological and psychiatric disorders (Pergola et al., 2018). Animal studies indicate the presence of a frequency-dependent gating mechanism within the MD-mPFC pathway. During low-frequency activity, strong feedforward inhibition confines excitation to a narrow temporal window, which may facilitate precise signal processing. In contrast, during high-frequency activity, the attenuation of feedforward inhibition and the broadening of the integration window may allow the sustained transmission of high-frequency MD activity to the mPFC, thereby supporting persistent activity loops between the MD and mPFC that are critical for higher cognitive functions such as short-term memory (Lee et al., 2020).

The impact of MD damage on memory ability is reversible. Animal experiments indicate that rats with MD lesions show significantly slower learning speeds for new tasks; however, after more extensive training, they achieve performance levels close to normal (Wolff et al., 2015). MD is essential for adaptive behavior that relies on current mental representations to guide actions based on stimulus-outcome associations, particularly in the absence of external sensory feedback. While MD lesions do not impair an animal’s ability to learn new associations in the presence of rewards, they severely compromise the ability to respond flexibly based solely on internal representations (Morceau et al., 2022), but it does not affect spatial working memory (Alcaraz et al., 2016). In rats, MD and mPFC form a functional circuit that is essential for supporting recognition memory requiring the integration of spatial associative information or temporal order, but is dispensable for simple single-item recognition memory (Cross et al., 2013). When rats learn instrumental action-outcome associations, task-relevant neural activity emerges in the MD-mPFC circuit. While both the MD and mPFC are involved in encoding reward feedback, they exhibit functional segregation in their oscillatory activity: MD shows enhanced theta activity, whereas mPFC exhibits increased gamma activity. Concurrently, inter-regional neural synchronization in the gamma band strengthens with learning, suggesting that functional coupling between the MD and mPFC plays a key role in integrating reward information to support learning (Yu et al., 2012).

### 3.2 Cognitive control

The role of MD and PFC in cognitive control are distinct: MD neurons are specialized for decision-making and response selection, whereas prefrontal neurons are specialized for preferentially encoding the environmental states upon which decisions are based (DeNicola et al., 2020). MD amplifies and maintains behaviorally relevant information within PFC. This process likely contributes to regulating mPFC neuronal responses to task-relevant information during goal-directed behavior. MD exerts a short-term influence on the expression of event-related activity in mPFC and a long-term effect on modulating how mPFC neurons respond to task-specific information (Mair et al., 2021). When task demands require rapid integration of visual and reward-based information, MDmc interacts with the cortex to coordinate neural communication within frontal regions (Perry et al., 2023). The reciprocal connectivity between MDl and the medial prefrontal cortex is involved in regulating adaptive control (Bruinsma et al., 2022). ATN and MD participate in attentional control through their connections with limbic structures. By virtue of its unique connectivity profile, MD maintains rule representations within the prefrontal cortex and modulates its functional microcircuitry, thereby supporting cognitive switching and behavioral flexibility. Neurons in MD can be activated by both rewarding and aversive stimuli, as well as by their predictive cues (Zhou et al., 2021).

MD plays a critical role in visuospatial attentional performance, as its reversible inactivation severely impairs attention. Importantly, this MD-mediated attentional function is not dependent on local muscarinic or nicotinic cholinergic receptor signaling, as blockade of these receptors does not replicate the severe behavioral deficits induced by inactivation. This suggests that MD supports prefrontal cortex-driven executive functions through non-cholinergic mechanisms (Mantanona et al., 2020). The activity state of MD, the rhythmic synchronization between the MD and prefrontal cortex, and the directional information flow from prefrontal cortex to MD collectively constitute a critical circuit predicting visual perceptual performance. MD serves as a key mediator, as a substantial portion of the prefrontal cortex’s influence on visual detection performance is indirectly exerted through MD. Manipulating MD activity was shown to diminish the direct effect of prefrontal cortex stimulation (Griffiths et al., 2022).

Both MD and mPFC are indispensable for rats to complete the odor span task, and they play dissociable roles in the regulation of foraging behavior (Scott et al., 2020). MD dynamically encodes chemosensory signals from the oral cavity. MD neurons respond not only to unimodal olfactory or gustatory stimuli but also integrate multisensory information, encoding both the palatability relevant to feeding decisions and established sensory associations (Fredericksen and Samuelsen, 2022).

### 3.3 Emotional responses

Neural projections from the anterior cingulate cortex to mediodorsal thalamic nucleus play a primary role in harm-avoidance behavior (Song et al., 2023) and emotional contagion responses (Zheng et al., 2020). Furthermore, the influence of MD on affect is also evident in responses to pain. In patients with chronic pain, there is a reduction in positive connectivity between MD and insula, hippocampus, and ventromedrontal prefrontal cortex, alongside an increase in negative connectivity between MD and the subgenual anterior cingulate cortex. These alterations are closely associated with the negative affect induced by chronic pain (Iwabuchi et al., 2023). Thalamic nuclei modulate both innate and conditioned fear responses. For instance, the tonic firing frequency of mediodorsal thalamic nucleus (MD) neurons correlates positively with the degree of fear extinction, and their distinct firing patterns may bidirectionally regulate the salience of fear-associated cues. Furthermore, MD is also involved in the transmission of social fear, potentially modulating the influence of social salience on fear responses (Zhou et al., 2021). Animal studies demonstrate that the cerebellum actively participates in the regulation of fear learning and extinction through multiple parallel pathways, including the fastigial nucleus-ventrolateral periaqueductal gray and fastigial nucleus-mediodorsal thalamic nucleus pathways (Urrutia Desmaison et al., 2023).

Mediodorsal thalamic nucleus (MD) serves not only as a critical hub for higher cognitive processes but also as a significant component of the central autonomic network, capable of modulating heart rate. Microstimulation of either the magnocellular (MDmc) or parvocellular (MDpc) subdivision of MD in anesthetized rhesus monkeys resulted in a significant increase in heart rate. Conversely, inducing cytotoxic lesions in these subregions led to a marked decrease in heart rate. MD shares extensive anatomical connections with established autonomic regulatory centers, including amygdala, periaqueductal gray, lateral hypothalamic area, and brainstem structures functionally associated with the limbic system, such as the locus coeruleus. Therefore, MD may act as a functional node that integrates information from cortical and subcortical sources to subsequently modulate cardiovascular output. Its widespread reciprocal connections with the prefrontal cortex underpin its central role in higher cognition, while concurrently enabling it to influence autonomic responses like heart rate. This suggests that MD may provide crucial interoceptive signals for cognitive and affective processes by modulating physiological states, thereby establishing a vital link between cognition, emotion, and physiology (Méndez et al., 2023).

## 4 MD in epilepsy: pathological changes and network role

### 4.1 Structural pathological alterations

Patients with mesial temporal lobe epilepsy (MTLE) exhibit significant thalamic atrophy (Keller et al., 2012). This atrophy is distributed in anterior thalamic nuclei, medial thalamic nuclei, and pulvinar, consistent with the lateralization of epilepsy. The volume of atrophy correlates with disease duration, and degree and distribution of pathological thalamic atrophy are related to pattern and extent of neocortical atrophy, suggesting that thalamus is an important hub within the TLE pathological network (Bernhardt et al., 2012). A postmortem study involving 24 epilepsy patients found that in cases of unilateral hippocampal sclerosis, there was a trend toward a significant reduction in neuronal density in the ipsilateral MD. Compared to other thalamic nuclei, MD ipsilateral to the hippocampal sclerosis showed evidence of neuronal loss, synapse reduction, or gliosis (Sinjab et al., 2013).

### 4.2 Functional connectivity abnormalities

Patients with epilepsy exhibit significant abnormalities in functional connectivity (FC) of MD. Ishizaki et al. used magnetoencephalography (MEG) to study the functional connectivity between MD and brain regions of default mode network (DMN) in patients with mesial temporal lobe epilepsy (MTLE). During the resting state, functional connectivity between MD and medial prefrontal cortex (mPFC) in MTLE patients was significantly increased in frequency bands ranging from Gamma to Ripple. Prior to the occurrence of interictal epileptic discharges (IEDs), functional connectivity between the MD and DMN-related brain regions was significantly decreased in the Ripple band (Ishizaki et al., 2023). Furthermore, in patients with MTLE, functional connectivity between mediodorsal thalamic nucleus and cingulate cortex is significantly reduced, and the degree of this reduction correlates with a higher frequency of epileptic seizures in this patients. Compared to healthy controls, patients with drug-resistant mesial temporal lobe epilepsy (mTLE) exhibit significantly reduced resting-state functional connectivity (RSFC) between the left magnocellular subdivision of MD (MDmc) and the cingulate cortex. The strength of RSFC between the MDmc and specific cingulate regions (right anterior midcingulate cortex, right dorsal posterior cingulate cortex) was significantly negatively correlated with the patients’ seizure frequency, meaning weaker connectivity was associated with a higher number of seizures per month. This inter-group difference in RSFC and its correlation with seizure frequency was specific to the MDmc and was not observed in other limbic system nuclei, such as the anterior thalamic nucleus. These findings suggest that MD, particularly its magnocellular subdivision (MDmc), plays a significant role in the pathological network of mTLE, and the weakening of its functional connectivity with the cingulate cortex may serve as a potential biomarker for disease severity (Jo et al., 2019). Piper et al. investigated alterations in the structural connectivity of MD in children with focal epilepsy. They found that in healthy children, the structural connectivity strength of MD decreased significantly with age, a trend that was absent in children with epilepsy. Compared to controls, all epilepsy patient groups (including temporal lobe epilepsy with hippocampal sclerosis, other temporal lobe epilepsies, and frontal lobe epilepsy) exhibited elevated structural connectivity strength of MD. While the structural connectivity strength of thalamic nuclei was generally higher in epilepsy patients compared to controls, this enhancing effect was most pronounced in mediodorsal nucleus (MD), centromedian nucleus (CM), and pulvinar (PUL), and least apparent in the anteroventral nucleus (AV) (Piper et al., 2026). Patients with generalized epilepsy exhibit both gray matter atrophy and elevated resting-state fMRI activity in MD. A disease-related network involving the sensorimotor cortex, supplementary motor area, anterior cingulate cortex, and thalamus, among other regions, is present in these patients. MD acts as a pivotal hub within this network, actively participating in its pathological architecture (Ji et al., 2025).

### 4.3 The role of MD in epileptic network dynamics

Wu et al. investigated the role of the mediodorsal thalamic nucleus in human seizure propagation using multi-site thalamic recordings. They found that MD was the earliest thalamic region involved during seizure spread in a subset of patients. For seizures sharing the same propagation network and seizure onset zone (SOZ), the specific thalamic subnucleus recruited (whether MD, ANT, or PUL) remained constant and exhibited a characteristic EEG signature. However, which particular thalamic subnucleus became involved first could not be reliably predicted based on the patient’s clinical semiology or the lobar localization of the SOZ (Wu et al., 2023). A study involving multi-site thalamic recordings in 23 patients with focal epilepsy demonstrated thalamic involvement in the early evolution of seizures, and the involvement of MD was closely associated with contralateral seizure spread. Furthermore, bilateral MD regions were often simultaneously engaged. Contralateral seizure spread was uncommon when connectivity to the ipsilateral MD was absent. In the majority of seizures that generalized to the contralateral hemisphere, the ipsilateral MD was involved prior to or concurrently with contralateral cortical sites (Marais et al., 2025). During epileptic seizures, high-frequency activity (low-voltage fast activity) can be detected in thalamic nuclei, including MD. Particularly at seizure onset, MD nucleus exhibits significant high-frequency activity. Abnormalities in EEG gamma activity within MD nucleus are associated with loss of consciousness. In the awake state, MD nucleus displays rhythmic gamma activity in the 30–40 Hz range. This activity ceases concurrently with the loss of consciousness when the seizure propagates to this region and reappears in synchrony with the recovery of consciousness. Abnormal discharges in MD nucleus correlate with unilateral abnormal discharges observed on the patient’s scalp EEG (Feys et al., 2025). In idiopathic generalized epilepsy, thalamus is a key nucleus driving generalized spike–wave discharges (GSWDs) from their initiation to propagation. This altered connectivity begins before onset of GSWDs and persists until the end of slow-wave activity (Zhang et al., 2015). Furthermore, sleep spindles are present in MD. Approximately 30% of thalamic sleep spindles are associated with interictal epileptiform discharges (IEDs). The frequency range of thalamic sleep spindles associated with IEDs is broader than that of normal sleep spindles, accompanied by a broadband increase in thalamic and cortical activity. The density of IEDs during MD sleep spindles correlates with the duration of the epilepsy condition (Szalárdy et al., 2021).

Animal experiments confirm that the thalamus promotes seizure evolution by prolonging the duration of excitatory drive through a divergent-convergent excitatory amplification system. This effect is operative not only in limbic system and focal epilepsies (Sloan et al., 2011) but has also been demonstrated in generalized epilepsies (Meeren et al., 2009). Young et al. analyzed multi-unit burst firing patterns in piriform cortex (PC) and mediodorsal thalamic nucleus in a model of absence epilepsy. They found clear early changes in spike-LFP phase-locking within the oscillatory band associated with communication between PC and MD, occurring prior to the onset of epileptic activity. This neural pathway may contribute to the initiation of absence seizures (Young et al., 2019). MD constitutes a critical component of the limbic epileptic circuitry. It is not only involved early during seizures, but its structural and functional alterations may also increase the excitability of epileptic networks, thereby playing a significant modulatory role in seizure dynamics. In rat models of kindling and chronic spontaneous epilepsy, MD is consistently activated from the earliest stages of seizures, with its electrical activity evolving synchronously with limbic structures such as the hippocampus. Chronically epileptic animals exhibit significant neuronal loss and regional atrophy in MD. Local injection of the anesthetic lidocaine into MD significantly shortened the afterdischarge duration of hippocampal seizures, whereas injections into the lateral thalamus were ineffective. This provides direct evidence that inhibiting activity in this region can modulate limbic seizures (Bertram et al., 2001). MD is not merely a pathway for the propagation of limbic seizures but an integral component of the primary epileptogenic circuit. It directly influences seizure duration and generalization by modulating the balance between excitatory and inhibitory neurotransmission. Injections of agents that enhance excitation (e.g., glutamate, AMPA, NMDA) or block GABAergic transmission (e.g., bicuculline) into the MD region significantly prolong seizure duration. Conversely, injection of a GABA agonist (muscimol) into the same region markedly shortens it. These effects are region-specific, as injections outside MD are ineffective. MD is the only thalamic nucleus from which hippocampal seizures can be directly induced, although its afterdischarge threshold is higher than that of traditional limbic structures like the amygdala and hippocampus. However, seizures induced from MD exhibit faster behavioral generalization (Bertram et al., 2008). Li et al. investigated the dynamic changes in phase locking value (PLV) of MD during epileptogenesis in a mouse model of amygdala electrical kindling. During the seizure period, MD, basolateral amygdala (BLA), and hippocampal CA1 region exhibited nearly simultaneous afterdischarges with similar waveforms. The duration of these discharges was positively correlated with seizure intensity. The timing of enhanced PLV between MD and other brain regions varied depending on the seizure stage. During partial seizures, the PLV increased sharply only afterseizure termination. In contrast, during secondary generalized seizures, the PLV was significantly enhanced beforeseizure termination. In the theta frequency band (4–8 Hz), the degree of PLV enhancement between the MD and other regions was positively correlated with seizure intensity. In partial seizures, the afterdischarge duration in the MD was occasionally longer than that in the BLA and CA1 regions (Li et al., 2016).

The roles of various thalamic nuclei in epileptic activity may be distinct. The pulvinar nucleus (PUL) is often the earliest thalamic site to be recruited during seizures, whereas the involvement of the anterior thalamic nucleus (ANT) is subject to individual variation (Marais et al., 2025; Wu et al., 2023). Activation of MD is strongly associated with contralateral propagation and generalization of epileptic seizures. In seizures that propagate contralaterally, the ipsilateral MD is recruited in over 96% of cases, and its involvement often precedes or coincides with that of the contralateral cortex (Marais et al., 2025). The centromedian nucleus (CM) serves as a critical functional connectivity hub within the network of idiopathic generalized epilepsy (IGE), and deep brain stimulation (DBS) targeting it has been clinically validated (Ji et al., 2025). Functional connectivity abnormalities of MD are closely associated with disease severity in mesial temporal lobe epilepsy (Ishizaki et al., 2023; Piper et al., 2026) (Table 1).

**Table 1:** Role of thalamic nuclei in epilepsy.

Thalamic nucleus	Primary network role and function	Key evidence and role in epilepsy	Predominant epilepsy syndrome/network
Mediodorsal thalamic nucleus (MD)	Cognitive control, working memory, emotional processing	Strongly associated with contralateral seizure generalization	Focal epilepsies with limbic-prefrontal network pathology
		Exhibits specific structural atrophy and functional connectivity abnormalities in mTLE, correlating with seizure frequency	
		Enhanced functional connectivity in high-frequency bands may reflect compensatory or aberrant network activity	
Anterior nucleus (ANT)	Papez circuit	Most involved	Focal epilepsies
Centromedian nucleus (CM)	Arousal/salience network node	Functional connectivity hub in the aberrant network of idiopathic generalized epilepsy	Idiopathic generalized epilepsy, Lennox–Gastaut syndrome
		CM-DBS has demonstrated clear efficacy in IGE and Lennox–Gastaut syndrome.	
Pulvinar (PUL)	Higher-order visual and attentional networks	Most involved	Epilepsies originating from the posterior cortex
	Relay for integrating information from posterior cortical regions	Exhibits widespread increased connectivity in patients with focal epilepsy	

## 5 Targeting MD for epilepsy therapy: exploration and attempts

Although electrophysiological recordings in both humans and experimental animals have revealed that MD may play a pivotal role in seizure initiation and propagation, interventional studies targeting MD have not yielded consistent therapeutic efficacy. Animal studies have shown that optogenetic inhibition of the pathway from mediodorsal thalamic nucleus to frontal cortex can suppress amygdala-kindled seizures. Inhibiting MD-PrL projection almost completely suppressed seizures, while inhibiting MD-IL projection reduced seizure severity but failed to shorten seizure duration. Inhibition of MD-OFC projection reduced seizures only with bilateral, high-dose stimulation, being ineffective with unilateral or low-dose stimulation. Inhibition of MD-ACC or MD-insular projections did not reduce seizures (Wicker et al., 2022). However, electrical stimulation-based neuromodulation of MD has not achieved similar effects, Wang et al. investigated the effects of deep brain stimulation (DBS) of MD on a rat model of amygdala electrical kindling. Experiments employed either low-frequency stimulation (LFS, 1 Hz) or high-frequency stimulation (HFS, 100 Hz), applied to the ipsilateral MD either after the kindling stimulus (post-treatment) or before it (preemptive). The results showed that neither LFS nor HFS DBS of MD, whether administered as a post-treatment or preemptively, significantly altered the acquisition rate of amygdala kindling or affected the afterdischarge threshold. Furthermore, stimulation of MD after full kindling was achieved also did not modify seizure expression parameters (Wang et al., 2010).

Although a growing number of researchers have recognized the important role of the MD in the initiation and evolution of epilepsy, there is currently no research investigating it as a target for deep brain stimulation (DBS) in the treatment of human epilepsy. In some case reports on CM-DBS for epilepsy treatment, the most effective stimulation sites were located ventral to the CM, within the ventral MD or the anteroventral pulvinar (Yang et al., 2024). This demonstrates the potential of MD as a target for neuromodulatory treatment of epilepsy. Employing a specifically optimized MP2RAGE sequence significantly enhances the visualization of human thalamic nuclei on 7 T MRI. This advancement not only allows for superior delineation of these nuclei in images but also provides a quantifiable improvement in the contrast-to-noise ratio (CNR). This technical advance holds significant implications for both research and clinical applications that require precise localization of deep brain structures (Marques and Gruetter, 2013). Jamiolkowski et al. reported a stereoelectroencephalography surgical strategy targeting MD, with no reported surgical complications, demonstrating the feasibility and safety of this technique (Jamiolkowski et al., 2025). Pantis et al. performed direct intracranial electrical stimulation of MD using both high and low frequency paradigms to examine its effects on conscious experience and brain connectivity. Stimulation of MD induced alterations in conscious experience, primarily in visceral, emotional, or somatosensory domains, often described as unpleasant sensations, without any lateralizing effects. MD stimulation revealed its strongest efferent connectivity to cingulate, insular, and PFC regions, all significantly stronger than that of medial temporal lobe structures. Notably, the afferent connectivity from medial temporal lobe and insula to MD was significantly stronger than the efferent connectivity from these areas, indicating a marked asymmetry in connectivity (Pantis et al., 2025). Furthermore, high-intensity, high-frequency stimulation of MD may potentially impair working memory (Peräkylä et al., 2017).

Although invasive DBS remains the gold standard for deep brain targeting, non-invasive techniques, such as temporal interference (TI) stimulation and low-intensity focused ultrasound (LIFU), provide a unique and lower-risk investigational pathway for exploring the therapeutic potential of MD prior to surgical intervention. The core technical principle of TI stimulation has been validated in patients with epilepsy, demonstrating its ability to non-invasively target and significantly suppress epileptiform activity in deep hippocampal structures (Missey et al., 2024). Furthermore, TI technology can be optimized for stimulating deep axonal tracts with greater focality by controlling the electric field orientation (Missey et al., 2021) or employing multipolar arrays (Missey et al., 2024), suggesting the future possibility of achieving selective modulation of specific output pathways from MD. Most directly, research using MRI-guided LIFU has proven that this technique can reversibly inhibit neuronal activity in the mediodorsal thalamic nucleus of non-human primates and alter its participation in cortical network information flow (Mishra et al., 2023), providing the most proximate proof-of-concept for “non-invasive targeting of MD.

## 6 Discussion

Structural atrophy, abnormalities in functional connectivity, and electrophysiological evidence of early activation and involvement in seizure propagation and generalization collectively establish the mediodorsal thalamic nucleus (MD) as a critical node within epileptic networks. The pivotal role of MD stems from its extensive connections with the limbic system, neocortex, and subcortical structures, forming a central hub for higher-order brain functions such as working memory, cognitive control, decision-making, attention, and emotional integration. Consequently, these pathological alterations within MD in patients with epilepsy may underlie the development of common comorbidities, including cognitive impairment, anxiety, and depression. Whether neuromodulation targeting MD can effectively treat these epilepsy-related comorbidities presents a promising avenue for future exploration.

Human epilepsy exhibits marked heterogeneity in both origin and phenotype. Epileptic seizures can originate from various cerebral lobes or even subcortical structures, and seizures from different foci often demonstrate substantial differences in their clinical semiology, electrophysiological patterns, and propagation pathways. These differences are manifested not only in observable behavior but are more profoundly reflected in the underlying alterations of brain networks. While a limited number of existing studies have explored the role of MD in epilepsy, they have predominantly focused on mesial temporal lobe epilepsy and simple motor seizures. Research has not yet extended to epilepsies originating from other lobes with which MD is closely connected, such as the frontal or occipital lobes, nor has it addressed complex motor seizures or psychic/psychosensory auras, which may be more intimately associated with MD function.

Studies in animal models have confirmed that precise optogenetic inhibition of specific pathways from MD to the prefrontal cortex can effectively control seizures. However, nonspecific electrical stimulation (e.g., deep brain stimulation) targeting MD nucleus itself has failed to yield stable therapeutic efficacy in either animal or human epilepsy. This discrepancy between a “well-defined mechanism” and “limited efficacy” highlights the current insufficient understanding of the distinct functional subregions of MD, its specific neural circuits, and its precise causal role in human epilepsy. While existing research has clearly delineated the topographic organization of MD-cortical fiber connectivity and the connectional differences among MD subregions, translating MD into an effective therapeutic target for epilepsy requires building upon this foundation. Future efforts should leverage high-resolution imaging and precise targeting technologies to explore deep brain stimulation strategies that specifically target either the critical white matter pathways or individual MD subregions, aiming to achieve precise interventions with higher efficacy and lower side effects. Furthermore, we note that in current studies, the selection of stimulation frequencies for deep brain stimulation (DBS) lacks sufficient experimental rationale. In animal experiments, frequencies of 1 Hz and 100 Hz have been used, while human studies have predominantly employed a single frequency of 145 Hz. Given that stereoelectroencephalography (SEEG) reveals an extremely wide frequency range for focal seizure onset (0.5–300 Hz), we propose that the DBS treatment frequency for patients with epilepsy should be individually tailored based on an analysis of the patient’s specific ictal onset pattern. This personalized approach may contribute to improved therapeutic efficacy. Moreover, non-invasive neuromodulation represents a significant direction for future research.

The patterns of involvement, network associations, and therapeutic potential of various thalamic nuclei in epilepsy are distinct, which determines the specific epilepsy syndrome types for which each may serve as an advantageous therapeutic target. Compared to PUL or ANT, the role of MD is more oriented towards seizure generalization. For seizure types prone to secondary generalization, inhibiting MD activity may be more effective in preventing seizure progression to bilateral convulsions than intervening at more upstream portal nodes. Furthermore, MD may be particularly suitable for epilepsy types accompanied by significant limbic-prefrontal network dysfunction.

Alterations in the structural and functional connectivity of MD correlate with seizure frequency and cognitive impairment, suggesting its potential as a biomarker. Future research should employ multimodal approaches to track the dynamic changes in MD connectivity, establishing quantitative associations with disease progression, treatment response, and cognitive outcomes. This will provide an objective basis for the personalized diagnosis and treatment of epilepsy. Although clinical recordings indicate early involvement of MD in seizures, it remains unclear whether its activity represents a “driving signal” for epileptogenesis or a “passive reverberation” of abnormal activity from other foci. This distinction requires validation in animal models utilizing combined optogenetic, chemogenetic, and electrophysiological recordings. Addressing these issues will not only deepen our fundamental understanding of epileptic network dynamics but will also directly facilitate the translation of novel therapeutic strategies targeting thalamocortical circuits from concept to clinic, ultimately offering new hope for patients with pharmacoresistant epilepsy.
